# A new CRISPR‐mediated *Apc* knockout allele leads to pyloric gland adenoma‐like gastric polyps in mice with C57BL/6;FVB/N mixed background

**DOI:** 10.1002/ame2.70002

**Published:** 2025-02-16

**Authors:** Sarp Uzun, Özge Özcan, Ayşenur Gök, Aynur Işık, Sinem Bakır, Ayşen Günel‐Özcan, İlyas Onbaşılar, Aytekin Akyol

**Affiliations:** ^1^ Hacettepe University Transgenic Animal Technologies Research and Application Center Ankara Turkey; ^2^ Department of Stem Cell Sciences Hacettepe University Graduate School of Health Sciences Ankara Turkey; ^3^ Department of Pathology Hacettepe University Faculty of Medicine Ankara Turkey; ^4^ Tumor Pathology Division Hacettepe University Cancer Institute Ankara Turkey; ^5^ Hacettepe University Molecular Pathology Research and Application Center Ankara Turkey; ^6^ Present address: Institute of Medical Genetics and Pathology University Hospital Basel Basel Switzerland

**Keywords:** adenomatous polyposis coli (APC), colorectal cancer, mouse model, pyloric gland adenoma

## Abstract

Adenomatous polyposis coli (*APC*) mutations are the most frequently identified genetic alteration in sporadic colorectal cancer (CRC) cases, and a myriad of genetically engineered *Apc*‐mutant CRC mouse models have been developed using various genetic manipulation techniques. The advent of the CRISPR/Cas9 system has revolutionized the field of genetic engineering and facilitated the development of new genetically engineered mouse models. In this study, we aimed to develop a novel *Apc* knockout allele using the CRISPR/Cas9 system and evaluate the phenotypic effects of this new allele in two different mouse strains. For this purpose, exon 16 of mouse *Apc* gene was targeted with a single‐guide RNA, and the mouse carrying an *Apc* frameshift mutation at codon 750 (Δ750) was chosen as the founder. The mutant FVB‐*Apc* ^Δ750^ mice were backcrossed with wild‐type C57BL/6 mice, and the phenotypic effects of the knockout allele were evaluated in F8‐FVB‐*Apc* ^Δ750^, F4‐B6;FVB‐*Apc* ^Δ750^, and F1‐B6;FVB‐*Apc* ^Δ750^ by a macroscopic and microscopic examination of the gastrointestinal system. The result showed that the mean polyp number was significantly higher in F4‐BL6;FVB‐*Apc* ^Δ750^ than in F8‐FVB‐*Apc* ^Δ750^. Intestinal polyposis was more prominent in F4‐BL6;FVB‐*Apc* ^Δ750^, whereas a higher number of colon polyps than intestinal polyps were observed in F8‐FVB‐*Apc* ^Δ750^. Additionally, F1‐BL6;FVB‐*Apc* ^Δ750^ mixed background mice developed gastric polyps that morphologically resembled the pyloric gland adenoma of humans. In conclusion, we developed a novel CRISPR‐mediated *Apc* knockout allele using two mouse strains. We showed that this allele can exert a strain‐specific effect on the phenotype of mice and can cause gastric polyp formation.

## Graphical abstract

The exon 16 of the mouse *Apc* gene was targeted with CRISPR/Cas9 system and a novel *Apc* knockout mouse model was generated by creating a frameshift mutation at codon 750 (*Apc* ^Δ750^). The polyp number, polyp distribution and the overall survival of mice carrying the *Apc* ^Δ750^ allele significantly differs between the F8‐FVB‐*Apc* ^Δ750^ and F4‐B6;FVB‐*Apc* ^Δ750^ mouse strains. F1‐BL6; FVB‐*Apc* ^Δ750^ mixed background mice develop gastric polyps which show morphologic similarity to human pyloric gland adenomas.

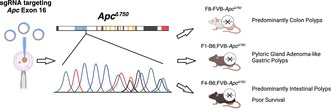

## 1 INTRODUCTION

Colorectal cancer (CRC) is the third most common cancer in both sexes and the second leading cause of death among all cancer types.^1^ Both environmental and genetic factors contribute to the development of CRC. Although most of the genetic alterations occur sporadically, 5% of all CRC patients suffer from hereditary CRC syndromes such as familial adenomatous polyposis (FAP) and Lynch syndrome.^2^ Since the first proposal of a genetic model for CRC, alterations in various genomic regions have been shown to be associated with CRC formation. Adenomatous polyposis coli (*APC*) is by far the most frequently altered gene during CRC progression, and its mutations are found in almost 80% of all CRC cases. Loss of function of APC protein leads to aberrant activation of the WNT pathway and triggers the neoplastic transformation of the normal colon epithelium.^3^

For almost 30 years, different mouse models of CRC have been defined to better understand the pathogenesis of this disorder and to develop effective treatment strategies against it. To date, various experimental strategies such as chemically induced carcinogenesis, transplantation‐based methods, and genetic engineering have been used to develop new CRC mouse models. With increasing knowledge of the genetic pathways altered in CRC, genetically engineered mouse models (GEMM) of CRC have become more widely used in experiments.^4^ Thus far, a myriad of CRC GEMMs have been developed, each of which can be classified into one of the four so‐called consensus molecular subtypes (CMS) of CRC. Because the vast majority of CRC patients carry a loss‐of‐function mutation in *APC*, the GEMMs developed by targeting this gene are the most frequently used models of CRC, which exhibit the features of canonical CMS2 tumors.^5^, ^6^

From the early 1970s to the present, various genetic manipulation strategies have been used to develop GEMMs.^7^, ^8^, ^9^ However, the introduction of the CRISPR/Cas9 system as a novel molecular tool for editing the mammalian genome has revolutionized the development of mouse disease models, and the widespread use of the CRISPR/Cas9 system by the scientific community has greatly facilitated the development of cancer GEMMs.^10^, ^11^, ^12^

In the present study, we aimed to develop a novel *Apc* knockout mouse model by targeting the genomic region encoding the seventh armadillo repeat (ARM) domain of the protein and to evaluate the effects of this new allele on the phenotype of two different mouse strains. Therefore, we created a frameshift mutation at codon 750 (*Apc* ^Δ750^) in the FVB/N mouse strain by targeting exon 16 with a single‐guide RNA (sgRNA) and transferred this allele to the C57BL/6 mouse strain by backcrossing. The results showed that the polyp number, polyp distribution, and overall survival of mice carrying the *Apc* ^Δ750^ allele differed significantly between the F8‐FVB‐*Apc* ^Δ750^ and F4‐B6;FVB‐*Apc* ^Δ750^ mouse strains. More surprisingly, F1‐BL6;FVB‐*Apc* ^Δ750^ mixed background mice developed gastric polyps, which morphologically resembled human pyloric gland adenomas (PGA). In conclusion, we have defined a novel mutant *Apc* allele that exhibits a strain‐specific phenotype in the mouse gastrointestinal system.

## 2 MATERIALS AND METHODS

### 2.1 Plasmid DNA construction, cloning of sgRNA, and embryo microinjection

The sgRNA oligo targeting exon 16 in *Apc* (target sequence: GTCTGCCATCCCTTCACGTTAGG ^13^) was cloned into the pX330‐U6‐Chimeric_BB‐CBh‐hSpCas9 (addgene plasmid 42230^14^) expression vector from the BbsI–BbsI restriction enzyme site downstream of the human U6 promoter. The final px300 product was transformed into JM109 (Promega, P9751) competent cells and plated on LB agar plates supplemented with 50 μg/mL of ampicillin. Single colonies were picked, plasmid isolation was performed using the GenElute HP Plasmid Miniprep Kit (Sigma, NA0150), and sgRNA insertion was verified by sequencing using the ABI 3130 Genetic Analyzer (Applied Biosystems, Foster City, CA). The plasmid (2.5–5 ng/μL) was injected into the pronucleus of FVB/N mouse embryos and transferred to the pseudopregnant FVB/N mice.

### 2.2 Superovulation of mice, embryo collection, and production of pseudopregnant mice

Female FVB/N mice (3–4 weeks old) were superovulated by intraperitoneal injection of pregnant mare serum gonadotropin (5 IU) on day 3 and human chorionic gonadotropin (5 IU) on day 1. Superovulated mice were mated with male FVB/N on day 1, and embryos were collected on day 0. Collected embryos were used for plasmid injection as described earlier. Pseudopregnant FVB/N mice were produced by mating them with surgically vasectomized male FVB/N mice the day before the microinjection.

### 2.3 Mouse colony management and breeding strategy

**FIGURE 1:**
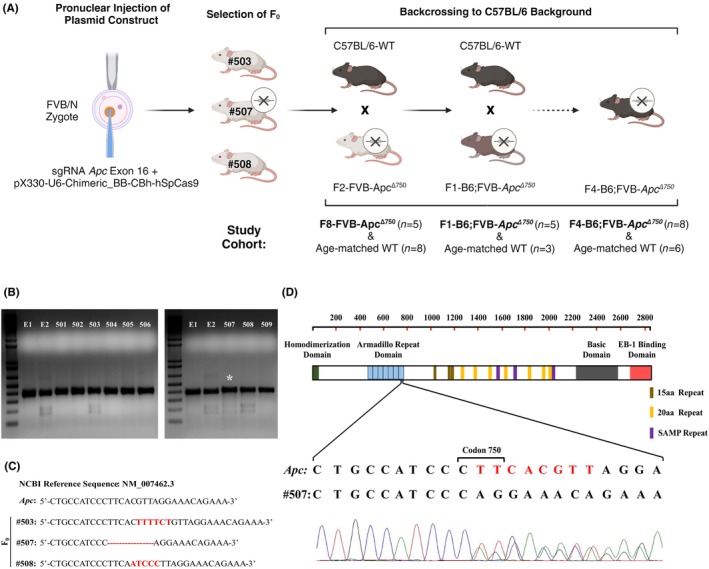
(A) Schematic illustration of experimental plan. T7E1 assay indicates the presence of heteroduplex structure in the PCR (polymerase chain reaction)–amplified region of E2, #503, #507, and #508 mice tail genomic DNA. (B) Asterix shows that #507 mice presented a weaker cleavage band. #503 founder has a six‐nucleotide insertion, #508 founder has a two‐nucleotide deletion and a five‐nucleotide insertion. (C) Only #507 founder carries a frameshift mutation due to an eight‐nucleotide deletion. (D) Representative illustration of the Apc protein and the site of frameshift mutation in the #507 founder mouse.

Transgenic founders on the FVB/N background were crossed with wild‐type (WT) mice, and experiments with FVB/N mice were performed using the F8 generation (F8‐FVB‐*Apc* ^Δ750^) and age‐matched WT control. By mating F2‐FVB‐*Apc* ^Δ750^ founder mice with WT C57BL/6 mice, the mutant allele was backcrossed to the C57BL/6 background. Experiments with C57BL/6 backcrosses were performed with F1 (F1‐B6;FVB‐*Apc* ^Δ750^) and F4 (F4‐B6;FVB‐*Apc* ^Δ750^) generations and age‐matched WT controls. The breeding strategy is shown in Figure 1A. All mice were housed in the specific pathogen‐free environment in an individually ventilated cage system under a 12‐h light–dark cycle at 22–23°C. Standard diet and water were provided ad libitum. All experimental procedures were performed according to the regulations of Hacettepe University Animal Experimentations Local Ethics Board (approval number: 2015/72) (Supplementary File).

### 2.4 Mutation analysis of *Apc* allele and sequencing

Mouse genomic DNAs were obtained from tail biopsies using the salting out method with slight modifications.^15^ The mutation site at *Apc* exon 16 was amplified with polymerase chain reaction (PCR) using Q5 High‐Fidelity DNA Polymerase (NEB, M0491S) with primers Apc_G‐F and Apc_G‐R, yielding a 397‐bp WT PCR product. PCR purification was performed using the GeneJET PCR Purification Kit (Thermo Fisher, K0702). T7 Endonuclease 1 (NEB, M0302) was used according to the manufacturer's instructions to determine the mismatched DNA samples. The PCR products were sequenced on an ABI 3130 Genetic Analyzer. After the determination of the mutation site in exon 16, Apc_Fmut primer was designed to recognize the mutation site. Genotyping was performed by multiplex PCR using primers Apc_F, Apc_R, and Apc_Fmut, which yielded a 268‐bp WT PCR product and a 185‐bp mutant PCR product (all primer sequences and PCR conditions are presented in Table S1).

### 2.5 Histologic and immunohistochemical analyses

Dissected gastrointestinal tissues were cut to open lengthwise, and polyps were counted under a Zeiss Stemi 305 microscope. The position of each polyp was marked on a sheet with the scheme of the mouse gastrointestinal tract, and the largest diameter of the polyps was measured with a scale. Then the polyps were divided into three categories based on their largest diameter: <2, 2–5, and >5 mm. For histologic analysis, tissues were fixed in 10% phosphate‐buffered formaldehyde and stained with hematoxylin and eosin. Immunohistochemical staining for β‐catenin was performed as previously described.^16^ The antibody against β‐catenin (BD Biosciences, 610154) was used at the final dilution of 1:1.000.

### 2.6 Statistical analysis

Descriptive analyses are presented as mean and standard error of the mean (SEM). The difference in total polyp count between mutant and matched WT mice was compared using Student's *t*‐test. The effect of the *Apc* ^*Δ750*^ allele on the survival of F4‐B6;FVB mice was analyzed using the Kaplan–Meier method, and the survival curves for time‐to‐event measures were compared using the log‐rank test. A *p*‐value <0.05 was used to infer statistical significance. Power estimates were not used. GraphPad Prism, version 9.2.0, for Windows (GraphPad Software, San Diego, CA, USA) was used for statistical analysis. Figures were created using BioRender (https://www.biorender.com/) and Inkscape, version 1.1.1.

## 3 RESULTS

### 3.1 Production of FVB/N mice carrying an *Apc* truncation mutation at codon 750 ( *Apc* ^Δ750^ ) using CRISPR/Cas9

We designed an sgRNA targeting the seventh ARM domain of *Apc* and cloned this sequence into the plasmid expressing mammalian Cas9. The plasmid was then injected into the pronuclei of fertilized FVB/N oocytes, and 601 one‐cell embryos were transferred to the surrogate mothers. A total of 11 mice were born alive, 2 of them (E1 and E2) died 2 days after birth, and the remaining 9 mice survived. Target region amplification and subsequent T7E1 assay revealed cleavage bands of ~200 bp in E2, #503 (female), #507 (male), and #508 (female) mice (Figure 1B). Three mice (#503, #507, #508) were selected as founders and crossed with WT FVB/N mice to obtain the F1 generation; 1 of 10 mice from #503 and 2 of 8 mice from #508 exhibited cleavage bands with the T7E1 assay. To confirm whether these three F1 mice carry a mutant *Apc* allele, the target region was sequenced, and all three mice were found to have an inframe mutation of *Apc* exon 16. In contrast to #503 and #508, only 2 of 56 mice (#587 and #603) from #507 had cleavage bands with the T7E1 assay, and sequencing of the target region showed that both these mice carried a mutant *Apc* allele with an 8‐bp deletion, resulting in a frameshift mutation in exon 16 (Figure 1C). The deletion mutation starts at codon 750 (*Apc* ^Δ750^) and leads to a stop codon formation at the codon 758. The frameshift mutation results in the formation of a truncated *APC* protein from the seventh ARM domain (Figure 1D).

### 3.2 *Apc* ^Δ750^ allele shows strain‐specific phenotype in the gastrointestinal system of F8‐FVB‐ *Apc* ^Δ750^ and F4‐BL6;FVB‐ *Apc* ^Δ750^ mice

**FIGURE 2:**
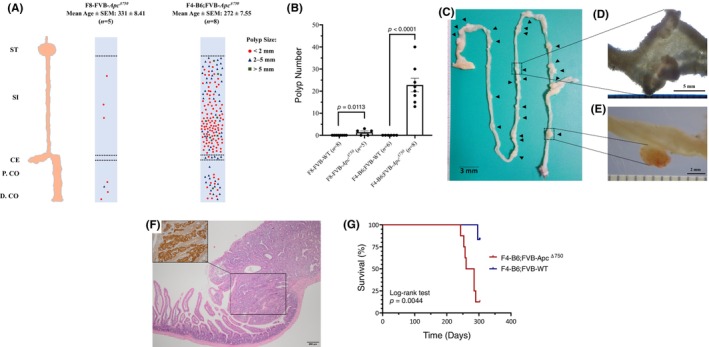
Polyp distribution of F8‐FVB‐*Apc* ^Δ750^ and F4‐B6;FVB‐*Apc* ^Δ750^ mice. Polyps from all mice in each cohort are represented in a single scheme by circles (<2 mm diameter), triangles (2–5 mm diameter), and squares (>5 mm diameter). (A) Stomach and proximal colon are spared from polyp in both strains. ST: Stomach, SI: Small intestine, CE: Cecum, P.CO: Proximal colon, D.CO: Distal colon. Polyp number comparison between F8‐FVB‐*Apc* ^Δ750^ mice (*n* = 5) and matched control mice (*n* = 8); F4‐B6;FVB‐*Apc* ^Δ750^ mice (*n* = 8) and matched control mice (*n* = 6). (B) Statistical analysis using 2‐tailed *t*‐test. Macroscopic image of the gastrointestinal system of an F4‐B6;FVB‐*Apc* ^Δ750^ mouse. (C) Arrowheads show the intestinal and colon polyps. (D) Microscopic image of an intestinal polyp in F4‐B6;FVB‐*Apc* ^Δ750^ mouse. (E) Microscopic image of a colon polyp in F4‐B6;FVB‐*Apc* ^Δ750^ mouse. H&E (hematoxylin and eosin) demonstrates an adenomatous polyp in the small intestine of an F4‐B6;FVB‐*Apc* ^Δ750^ mouse. (F) Inset indicates the nuclear β‐catenin accumulation in neoplastic cells. Overall survival of F4‐B6;FVB‐*Apc* ^Δ750^ mice (*n* = 8) and matched control mice (*n* = 6). (G) Statistical analysis using log‐rank test.

The two F1 mice (#587 and #603) were crossed with WT FVB/N mice to produce an FVB‐*Apc* ^Δ750^ mouse line, and we examined the phenotypic effects of the allele in F8‐FVB‐*Apc* ^Δ750^ mice. All F8‐FVB‐*Apc* ^Δ750^ mice survived until the termination of the cohort at a mean age of 331 days. The F8‐FVB‐*Apc* ^Δ750^ mice produced a small number of polyps (mean ± SEM: 1.40 ± 0.60), which were primarily localized in the distal colon (Figure 2A,B). All polyps except one in the distal colon had a maximum diameter of <2 mm. To evaluate if this mild phenotype in the FVB/N mice was a strain‐specific effect of the allele, we backcrossed the *Apc* ^Δ750^ allele to the C57BL/6 background. After multiple crosses with WT C57BL/6 mice, we performed phenotyping on F4‐BL6;FVB‐*Apc* ^Δ750^ mice. In significant contrast to the FVB/N strain, F4‐BL6;FVB‐*Apc* ^Δ750^ mice were characterized with severe polyposis (mean ± SEM: 22.75 ± 3.10), and the polyps were distributed throughout the gastrointestinal system except for the stomach and proximal colon (Figure 2A,B). Intestinal polyp formation was significantly more pronounced in the F4‐BL6;FVB‐*Apc* ^Δ750^ mice compared to the F8‐FVB‐*Apc* ^Δ750^ mice; nevertheless, all F4‐BL6;FVB‐*Apc* ^Δ750^ mice had at least one colon polyp (Figure 2C). The intestinal and colon polyps had different macroscopic morphology. Intestinal polyps exhibited a disc‐shaped morphology (Figure 2D), whereas the colon polyps were pedunculated and well vascularized (Figure 2E). Intestinal and colon polyps exhibited an adenomatous morphology, and they exhibited strong β‐catenin accumulation in the nuclei of neoplastic cells (Figure 2F). All but one of the F4‐BL6;FVB‐*Apc* ^Δ750^ mice died or were euthanized before 300 days due to the rapid deterioration of health, and survival analyses showed that the *Apc* ^Δ750^ allele was significantly associated with poor prognosis in F4‐BL6;FVB‐*Apc* ^Δ750^ mice (Figure 2G). In addition to polyp formation in the gastrointestinal system, two F8‐FVB‐*Apc* ^Δ750^ mice developed subcutaneous nodules (data not shown).

### 3.3 F1‐B6;FVB‐ *Apc* ^Δ750^ mice develop PGA‐like gastric polyps

**FIGURE 3:**
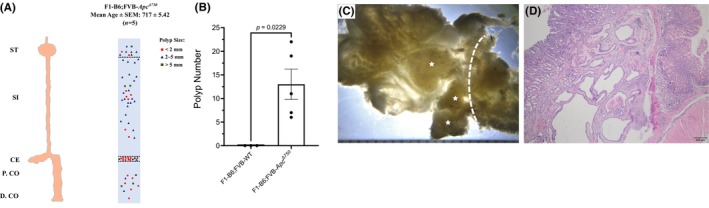
Polyp distribution of F1‐BL6;FVB‐*Apc* ^Δ750^ mice. (A) Polyps from all mice in F1‐BL6;FVB‐*Apc* ^Δ750^ mice cohort are represented in a single scheme by circles (<2 mm diameter), triangles (2–5 mm diameter), and squares (>5 mm diameter). ST: Stomach, SI: Small intestine, CE: Cecum, P.CO: Proximal colon, D.CO: Distal colon. (B) Polyp numbers in F1‐BL6;FVB‐*Apc* ^Δ750^ and wild‐type control mice. Microscopic image of the gastroduodenal junction of an F1‐BL6;FVB‐*Apc* ^Δ750^ mouse. Stars show gastric polyps that are situated at the pylorus. (C) Dashed line indicates the gastroduodenal junction. (D) H&E (hematoxylin and eosin) demonstrates a gastric polyp that morphologically resembles a human pyloric gland adenoma.

Because F8‐FVB‐*Apc* ^Δ750^ and F4‐BL6;FVB‐*Apc* ^Δ750^ mice exhibited distinct phenotypic features with the *Apc* ^Δ750^ allele, we hypothesized that F1‐BL6;FVB‐*Apc* ^Δ750^ mixed background mice might exhibit characteristics of both strains. Indeed, the distribution of polyps in the gastrointestinal system of F1‐BL6;FVB‐*Apc* ^Δ750^ mice showed similar features of both strains: the proximal colon and ileum remained tumor free as we observed in F8‐FVB‐*Apc* ^Δ750^ mice; however, intestinal polyposis was more prominent in F4‐BL6;FVB‐*Apc* ^Δ750^ mice (Figure 3A). All F1‐BL6;FVB‐*Apc* ^Δ750^ mice in our cohort could live more than 700 days, and despite their long lifespan, they exhibited a milder phenotype with lower polyp numbers (mean ± SEM: 13.0 ± 3.21) compared to F4‐BL6;FVB‐*Apc* ^Δ750^ mice (Figure 3B). Surprisingly, four of five mice developed multiple gastric polyps, which were all localized to the antrum and pylorus (Figure 3C). Microscopic evaluation of the gastric polyps revealed that their morphological appearance closely resembled human PGA (Figure 3D).

## 4 DISCUSSION

In the present study, we developed a new *Apc* knockout allele using the CRISPR/Cas9 system and evaluated the phenotypic effects of this allele using two different mouse strains. We showed that *Apc* knockout mice exhibited gastrointestinal polyposis in both strains but with a strain‐specific phenotype. More interestingly, we found that F1‐BL6;FVB‐*Apc* ^Δ750^ mixed background mice could develop gastric polyps with morphology similar to human PGA.

*Apc* knockout mouse models are widely used in CRC research. The most commonly used mouse model of CRC is multiple intestinal neoplasia (or *Apc* ^Min/+^), which was first described in 1990. This mouse model was developed using *N*‐ethyl‐*N*‐nitrosourea (ENU)–induced chemical mutagenesis, leading to a truncation mutation of *Apc* gene at codon 850. *Apc* ^Min/+^ mice on C57BL/6 background exhibit predominantly small intestinal polyps, and these mice can survive rarely beyond 120 days.^17^ In our study, F4‐BL6;FVB‐*Apc* ^Δ750^ mice exhibited a strong phenotypic resemblance to the classical *Apc* ^Min/+^ mouse model. They developed intestinal polyps localized predominantly in the distal half of the small intestine. All F4‐BL6;FVB‐*Apc* ^Δ750^ mice exhibited a few colonic polyps (mean ± SEM: 2.75 ± 0.97), and they were mainly localized in the distal colon by sparing the proximal colon. F4‐BL6;FVB‐*Apc* ^Δ750^ mice presented a better prognosis compared to classical *Apc* ^Min/+^ mice; however, it should also be considered that the study was carried out with the F4 generation, and the phenotype may become more pronounced after further backcrossing with WT C57BL/6 mice.

To date, different intestinal polyposis mouse models have been developed by targeting the *Apc* gene to mimic the *Apc* mutant CRCs. In some of these models, the ARM domain of *Apc* was targeted, resulting in the expression of shorter *Apc* protein compared to the *Apc* ^Min/+^ model.^18^ These models share many phenotypic features with *Apc* ^Min/+^ mice, including embryonic lethality in the homozygous state, anemia, and predominant intestinal polyposis. Although the polyp numbers vary between models, they show similarities to those seen in *Apc* ^Min/+^. However, in an exceptional study, Oshima et al. developed a mouse model with a frameshift mutation at codon 716 (*Apc* ^Δ716^), which produced almost three times more intestinal polyps than the other models targeting the ARM domain.^19^ In our *Apc* ^Δ750^ model, we created a truncation mutation at codon 750 which corresponds to the seventh ARM domain of the Apc protein. In contrast to Oshima et al., our F4‐BL6;FVB‐*Apc* ^Δ750^ model developed a similar number of polyps to the *Apc* ^Min/+^ model and supported the current literature that the truncation mutations at the ARM region mostly develop mouse models phenotypically similar to the classical *Apc* ^Min/+^. Mouse models of *Apc*, particularly the *Apc* ^Min/+^, are valuable models to study the contribution of environmental factors, microbiota, and other altered genes to CRC pathogenesis. In addition, these models are widely used in preclinical studies to develop novel chemopreventive strategies against CRC.^20^, ^21^ Therefore, our model has the potential to be a useful GEMM that can be used in similar study designs.

It is well known that the strain‐specific expression of different genes can lead to phenotypic differences in mouse models.^22^ The influence of modifier genes was also observed in the *Apc* ^Min/+^ mouse model after backcross experiments. Moser et al. backcrossed Δ850 allele from BL6 to AKR background and observed a significant decrease in the number of intestinal tumors.^23^ Further investigation of the potential genetic markers resulted in the discovery of a *modifier of min‐1 (Mom‐1)* locus and was found to be responsible for most of the variation in tumor number.^24^ To date, several different *Mom* loci have been identified, and their strain‐specific effects are under active assessment.^25^ To evaluate the influence of mouse strain on the *Apc* ^Δ750^ phenotype, we transferred the mutant allele to C57BL/6 mice from F2‐FVB‐*Apc* ^Δ750^ mice by backcrossing. First, we observed that *Apc* ^Δ750^ phenotype was seen in all offspring by exhibiting full penetrance as in the *Apc* ^Min/+^ model.^23^ Then, we compared the gastrointestinal polyp number, polyp size, and polyp distribution of two *Apc* ^Δ750^ mouse strains and showed that FVB/N mice developed significantly fewer polyps than BL6 mice. The resistance of the FVB/N background to polyp formation has also been demonstrated in *Apc* ^Min/+^ mice by other studies, suggesting that this strain carries dominantly acting modifier genes that affect tumor development.^26^, ^27^ Furthermore, in these studies, treatment of FVB‐*Apc* ^Min/+^ mice with chemical agents/carcinogens like ENU and 2‐amino‐1‐methyl‐6‐phenylimidazo [4,5‐b]pyridine significantly increased polyp number. Therefore, our *Apc* ^Δ750^ mouse model on FVB/N background can also be used as an in vivo model to test the carcinogenic potential of certain chemicals, but this should be validated in further studies.

*Apc* knockout mouse models are primarily characterized by intestinal tumor formation, which might be accompanied by a small number of colonic polyps and aberrant crypt foci.^5^ In a study, the stomach of 24‐week‐old *Apc* ^Min/+^ mice was morphologically evaluated. They found that the forestomach of *Apc* ^Min/+^ mice was heavily infiltrated with lymphocytes, and it was associated with epithelial proliferation. Interestingly, they also observed that in contrast to the forestomach and corpus, the antral mucosa of the mice contained irregularly branching glands lined by atypical columnar cells.^28^ Our findings supported this observation and indicated that the presence of the *Apc* ^Δ750^ allele in F1‐hybrid mice could lead to gastric polyp formation in the antral and pyloric regions. More interestingly, the histologic evaluation of these tumors revealed their morphological resemblance to PGA of humans. PGAs are rare neoplasia that primarily arise from the stomach. Histologically, PGAs consist of pyloric gland‐like epithelium with low columnar cells with typical ground glass cytoplasm. They are found in 6% of all FAP patients and make up 15% of all adenomas in FAP.^29^, ^30^ In our study, PGAs were observed only in four of five F1‐BL6;FVB‐*Apc* ^Δ750^ mixed background mice euthanized at ~700 days of age and were not seen in F8‐FVB‐*Apc* ^Δ750^ or F4‐B6;FVB‐*Apc* ^Δ750^ mice. Therefore, this may indicate that the development of gastric polyps requires a longer time period than intestinal and colonic polyps in the context of *Apc* loss of function.

There are several limitations in our study. First, we backcrossed the *Apc* ^Δ750^ allele to the C57BL/6 background and performed the experiments in F4‐BL6;FVB‐*Apc* ^Δ750^ mice, which are expected to carry more than 90% of the original C57BL/6 genotype. Although this model recapitulates most of the phenotypic features of the *Apc* ^Min/+^ mouse on the C57BL/6 strain, further backcrossing with inbred strains may make the phenotype more pronounced. Second, we observed that the F1‐BL6;FVB‐*Apc* ^Δ750^ mixed background mice had gastric polyps at ~700 days of age. Because almost all F4‐BL6;FVB‐*Apc* ^Δ750^ mice died before 300 days and F8‐FVB‐*Apc* ^Δ750^ mice were euthanized ~300 days, we could not follow these two cohorts for 700 days as we did with the mixed background mice. Therefore, we cannot claim that gastric polyposis is a specific feature of mixed background mice, and further long‐term follow‐up studies comparing the polyp distribution of FVB‐*Apc* ^Δ750^ and F1‐BL6;FVB‐*Apc* ^Δ750^ mixed background mice should be carried out.

In conclusion, we developed a new *Apc* knockout allele by targeting the seventh ARM domain and characterized the phenotype in two different mouse strains. We showed that F8‐FVB‐*Apc* ^Δ750^ mice developed fewer gastrointestinal polyps and had a better overall survival than F4‐BL6;FVB‐*Apc* ^Δ750^ mice. In addition, we found that F1‐BL6;FVB‐*Apc* ^Δ750^ mixed background mice could develop gastric polyps that morphologically resemble the gastric PGA of humans.

## AUTHOR CONTRIBUTIONS

**Sarp Uzun:** Conceptualization; data curation; formal analysis; methodology; software; visualization; writing – original draft; writing – review and editing. **Özge Özcan:** Conceptualization; data curation; formal analysis; methodology; visualization; writing – original draft; writing – review and editing. **Ayşenur Gök:** Data curation; formal analysis; methodology. **Aynur Işık:** Data curation; formal analysis; methodology. **Sinem Bakır:** Data curation; formal analysis; methodology. **Ayşen Günel‐Özcan:** Conceptualization; project administration; supervision; writing – review and editing. **İlyas Onbaşılar:** Conceptualization; project administration; supervision; writing – review and editing. **Aytekin Akyol:** Conceptualization; data curation; formal analysis; funding acquisition; investigation; methodology; project administration; resources; supervision; writing – original draft; writing – review and editing.

## FUNDING INFORMATION

This work was supported by the Scientific and Technological Research Council of Turkey (TUBITAK) under the 1001 program with project number SBAG‐215S926 to Aytekin Akyol.

## CONFLICT OF INTEREST STATEMENT

The authors declare that they have no conflicts of interest.

## ETHICAL STATEMENT

All experimental procedures were carried out according to the regulations of Hacettepe University Animal Experimentations Local Ethics Board (approval number: 2015/72).

## Supporting information


Supplementary Table 1.

Data S1.
