# Supplementary material for: A new CRISPR‐mediated Apc knockout allele leads to pyloric gland adenoma‐like gastric polyps in mice with C57BL/6;FVB/N mixed background
Source: Animal Model Exp Med. 2025 Feb 16;8(5):922–9. doi: 10.1002/ame2.70002 (PMC12107358; doi:10.1002/ame2.70002)
Supplement: Supplementary file 1 — Supplementary Table 1. [file AME2-8-922-s002.docx]

**Supplementary Table 1. Primer sequences used for genotyping**

| **Primer Name** | **Primer Sequence (5´- 3´)** | **Annealing Temperature/**  **PCR Cycles** |
| --- | --- | --- |
| Apc_G_F | CATGTGGAACTTTGTGGAATC | 60° C / 39 |
| Apc_G_R | TCTGACCTACTATCATCATGTCG |  |
| APC_F | CAGCAGCTTTAAGGAATCTCA | 59° C / 35 |
| APC_R | TCTGACCTACTATCATCATGTCG |  |
| APC_Fmut | GTCTGCCATCCCAGGAAA |  |
