# Supplementary material for: A new CRISPR‐mediated Apc knockout allele leads to pyloric gland adenoma‐like gastric polyps in mice with C57BL/6;FVB/N mixed background
Source: Animal Model Exp Med. 2025 Feb 16;8(5):922–9. doi: 10.1002/ame2.70002 (PMC12107358; doi:10.1002/ame2.70002)
Supplement: Supplementary file 2 — Data S1. [file AME2-8-922-s001.pdf]

HACETTEPE UNIVERSITY
ANIMAL EXPERIMENTATIONS
LOCAL ETHICS BOARD

O6JOO Sıhhiye Ankara
Telephone: 0 (312) 305 1090 i08Z - Fax: 0 (312) 310 0580
www.etikkurul.hacel tepe.edu.tr/index_hdk.php

Number: B.30.2.HAC.0.05.06.00/

Issue: 52338575 t\}

ANIMAL EXPERIMENTATIONS LOCALETHICS BOARD DECISION

MEETING DATE NUMBER OF MEETINGS	27.08.2015 (THURSDAY) 2015/07
FILE RECORD NUMBER	2015/72
**DECISION NUMBER**	2015/72 - 03
RESEARCH DIRECTOR	Assoc. Prof. Dr. Aytekin AKYOL
RESEARCHER RESPONSIBLE FOR ANIMAL EXPERIMENTS	Assoc. Prof. Dr. Aytekin AKYOL, Assoc. Prof. Dr. İlyas ONBAŞILAR and Bio. Aynur IŞIK
assistant researchers **species** and number of animals approved	Assoc. Prof. Dr. Ayşen Günel ÖZCAN 280 FVB/N mice

The study titled ***"Generation of Intestinal Polyposis In Mice with CRISPR/Cas9 System" with the*** registration number 2015/72, in which Assoc. Prof. Dr. Aytekin AKYOL, one of the faculty members of the Department of Medical Pathology of the Faculty of Medicine of our university, was found appropriate according to the Directive of the Animal Experiments Local Ethics Committee and it was decided to be approved unanimously. Board member Assoc. Prof. Dr. Aytekin AKYOL did not participate in the discussion and voting due to conflict of interest.

The principal investigator is obliged to notify the Ethics Committee of the start date of the experiments

[redacted]
Prof. Dr. Sema ÇALIŞ Chair of the Ethics Committee

animal experiments local ethics committee agenda - signature circulars meeting date

	27.08.2015 (THURSDAY)
NUMBER	OF MEETINGS 2015/07
MEETING TIME	: 13.30

[redacted] Prof. Dr. Sema Calis (Chair)	[redacted] Prof. Dr. Nüket Örnek Büken (Member)	[redacted] Prof. Dr. M. Yıldırım Sara [redacted]
[redacted] Prof. Dr. Neslihan H. Dikmenoglu Falkmarken (Üye)	[redacted] Prof. Dr. Belgin Can (Membe r)	[redacted] Prof. Dr. Mehmet Ali Onur (Member)
[redacted] Assoc. Prof. Hakan EL (Member)	[redacted] Assoc. Prof. Dr. Aytekin Akyol (Member)	[redacted] Assoc. Prof. Dr. Ersoy KONAŞ (Member)
[redacted] Assoc. Prof. Dr. İlyas Onbaşılar (Member)	[redacted] Doç. Dr. M , f Çetinrock (Me mber)	[redacted] Assist. Assoc. Prof. Dr. Banu Cahide Tel (Member)
[redacted] Mevlüt Öksüzoğlu (Member)	[redacted] Lawyer Yasemin Özselçuk (Member)	
